# ALDH2 contributes to melatonin-induced protection against APP/PS1 mutation-prompted cardiac anomalies through cGAS-STING-TBK1-mediated regulation of mitophagy

**DOI:** 10.1038/s41392-020-0171-5

**Published:** 2020-07-24

**Authors:** Shuyi Wang, Lin Wang, Xing Qin, Subat Turdi, Dongdong Sun, Bruce Culver, Russel J. Reiter, Xiaoming Wang, Hao Zhou, Jun Ren

**Affiliations:** 1 Department of Emergency, Shanghai Tenth People’s Hospital, Tongji University School of Medicine, Shanghai, 200072 China; 2 Center for Cardiovascular Research and Alternative Medicine, University of Wyoming College of Health Sciences, Laramie, WY 82071 USA; 3 Department of Cardiology and Shanghai Institute of Cardiovascular Diseases, Zhongshan Hospital Fudan University, Shanghai, 200032 China; 4 Department of Geriatrics, Xijing Hospital, The Air Force Military Medical University, Xi’an, China; 5 Department of Cardiology, Xijing Hospital, The Air Force Military Medical University, Xi’an, 710032 China; 6 Department of Cellular and Structural Biology, UT Health San Antonio, San Antonio, TX USA; 7 Chinese PLA General Hospital, Medical School of Chinese PLA, Beijing, 100853 China

**Keywords:** Cardiology, Neurological disorders

## Abstract

Ample clinical evidence suggests a high incidence of cardiovascular events in Alzheimer’s disease (AD), although neither precise etiology nor effective treatment is available. This study was designed to evaluate cardiac function in AD patients and APP/PS1 mutant mice, along with circulating levels of melatonin, mitochondrial aldehyde dehydrogenase (ALDH2) and autophagy. AD patients and APP/PS1 mice displayed cognitive and myocardial deficits, low levels of circulating melatonin, ALDH2 activity, and autophagy, ultrastructural, geometric (cardiac atrophy and interstitial fibrosis) and functional (reduced fractional shortening and cardiomyocyte contraction) anomalies, mitochondrial injury, cytosolic mtDNA buildup, apoptosis, and suppressed autophagy and mitophagy. APP/PS1 mutation downregulated cyclic GMP-AMP synthase (cGAS) and stimulator of interferon genes (STING) levels and TBK1 phosphorylation, while promoting Aβ accumulation. Treatment with melatonin overtly ameliorated unfavorable APP/PS1-induced changes in cardiac geometry and function, apoptosis, mitochondrial integrity, cytosolic mtDNA accumulation (using both immunocytochemistry and qPCR), mitophagy, and cGAS-STING-TBK1 signaling, although these benefits were absent in APP/PS1/ALDH2^−/−^ mice. In vitro evidence indicated that melatonin attenuated APP/PS1-induced suppression of mitophagy and cardiomyocyte function, and the effect was negated by the nonselective melatonin receptor blocker luzindole, inhibitors or RNA interference of cGAS, STING, TBK1, and autophagy. Our data collectively established a correlation among cardiac dysfunction, low levels of melatonin, ALDH2 activity, and autophagy in AD patients, with compelling support in APP/PS1 mice, in which melatonin rescued myopathic changes by promoting cGAS-STING-TBK1 signaling and mitophagy via an ALDH2-dependent mechanism.

## Introduction

Alzheimer’s disease (AD) is an irreversible neurodegenerative pathology commonly characterized by overt buildup of amyloid β-peptide (Aβ) and neurofibrillary tangles, which results in memory loss, cognitive impairment, and shortened lifespan.^1^ Although AD etiology is predominantly idiopathic, an autosomal dominant disorder elicited by mutant β-amyloid precursor protein (APP), presenilin 1 (PS1), or presenilin 2 (PS2) is believed to contribute to familial AD.^2^ Recent evidence supports a close association between AD and cardiac dysfunction.^1,3–5^ Considering the localization of the AD genes PS1 and PS2 in the heart,^6^ it is plausible that there is a role for mutations in PS1/PS2 in the elevated risk of cardiac dysfunction in AD patients.^7^ Earlier findings indicated a role of PS1/PS2 in the regulation of cardiovascular function, as PS1-deficient mice display septal defects and double outlet right ventricles.^8^ Likewise, PS2 contributes to cardiac excitation–contraction through direct coupling with the type 2 ryanodine receptor.^9^ Using mouse models that overexpress FAD-linked APP with Swedish mutation and deletion of exon 9 of PS1 (APPswe/PS1dE9), Aβ deposition is seen as early as 4–6 months of age in mice and full-blown Aβ-peptide deposition by 9 months in APPswe/PS1dE9 mice.^10^ Earlier findings from our own laboratory and others demonstrated pronounced cardiac anomalies in this murine model of AD.^11–13^ Although several scenarios, including oxidative stress and the cytotoxicity of Aβ, may play a role,^1,5^ the pathogenesis of AD-induced cardiac pathology remains poorly understood.

Melatonin (*N*-acetyl-5-methoxytryptamine) is a pleiotropic endogenous hormone that is mainly synthesized in the pineal gland and has proven benefits in aging-related neurodegenerative diseases, including d-galactose- and AD-induced memory or synaptic deficit.^14,15^ As a potent antioxidant, melatonin protects against a wide variety of cardiovascular and neurodegenerative diseases, as well as Aβ-induced neurotoxicity and mitochondrial damage.^16–20^ Given the well-established cardiovascular benefit of melatonin in the maintenance of cardiac geometry and function,^19,20^ this study examined the correlation between melatonin levels and cardiac anomalies in AD. Earlier evidence documented an essential role for mitochondrial aldehyde dehydrogenase (ALDH2) in an array of cardiovascular anomalies and neurodegenerative diseases, including AD,^21–32^ although controversy still exists with regard to ALDH2 polymorphisms and AD onset.^33,34^ Two forms of melatonin receptors, melatonin receptor subtype 1a or MTNR1A (MT1) and melatonin receptor subtype 1b or MTNR1B (MT2), are present and mediate melatonin-induced biological effects.^17,35^ The clinical observation of the present study revealed overt cardiac dysfunction in AD patients associated with low levels of circulating melatonin, ALDH2 activity, and autophagy. These data were supported by our experimental data showing that melatonin supplementation rescued APPswe/PS1dE9-induced cardiac anomalies with restored ALDH2 activity, mitochondrial ultrastructure, cytosolic mtDNA content, autophagy, and mitophagy. An obligatory role for ALDH2 was consolidated, as ALDH2 deletion nullified melatonin-mediated cardioprotection. Our findings failed to note any change in melatonin membrane receptor (MT1 or MT2) abundance in response to APP/PS1 mutation, melatonin supplementation, or ALDH2 ablation. However, the melatonin-mediated cardioprotective effects against APPswe/PS1dE9-induced cardiac anomalies were nullified by the nonspecific melatonin receptor antagonist luzindole, indicating a receptor-dependent mechanism for melatonin. Our findings also revealed an essential role for cyclic guanosine monophosphate-adenosine monophosphate synthase (cGAS)-stimulator of interferon genes (STING)-mediated regulation of autophagy, possibly through the mitochondrial chaperone molecule ALDH2-mediated control of cytosolic accumulation of mitochondrial DNA (mtDNA), which is an intrinsic trigger for cGAS-STING activation.^36^

## Results

### Echocardiographic function and circulating melatonin levels in AD patients

**Fig. 1:**
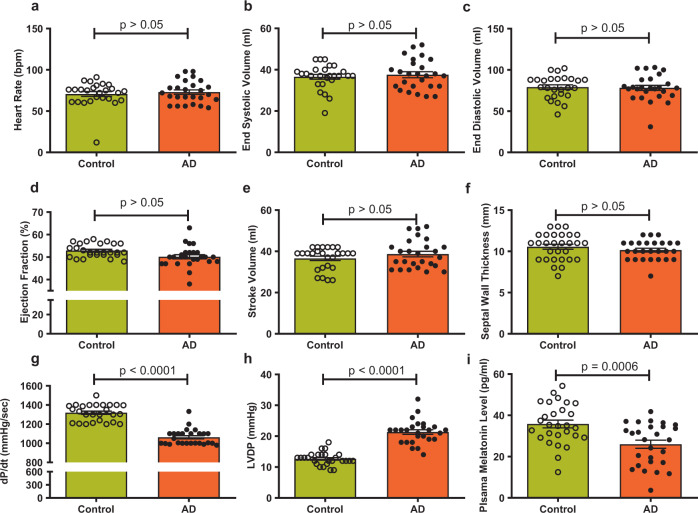
Heart function and melatonin levels in Alzheimer’s disease patients and age-matched controls. **a** Heart rate; **b** end systolic volume; **c** end diastolic volume; **d** ejection fraction (%); **e** stroke volume; **f** septum wall thickness; **g** dP/dT; **h** LVDP (left ventricular diastolic pressure); and **i** circulating melatonin concentration. The data are shown as the mean ± SEM, *n* = 26 individuals per group, and all *p*-values shown were calculated using Student’s *t*-tests

Cognitive scores confirmed the clinical diagnosis of AD in patients. There was no difference in age or gender distribution (Supplementary Table S1). Echocardiography examination showed that heart rate, end systolic volume, end diastolic volume, ejection fraction, stroke volume, and septal wall thickness were comparable between the two groups. However, the rate of pressure increase during ventricular contraction (dP/dt) was overtly lower with elevated left ventricular (LV) diastolic pressure in AD patients than those in healthy patients. These echocardiographic features were accompanied by overtly reduced circulating melatonin levels in AD patients (Fig. 1). These data revealed cardiac dysfunction in AD patients in association with reduced circulating melatonin levels.

### Effect of melatonin on APP/PS1-induced cognitive and cardiac contractile anomalies

**Fig. 2:**
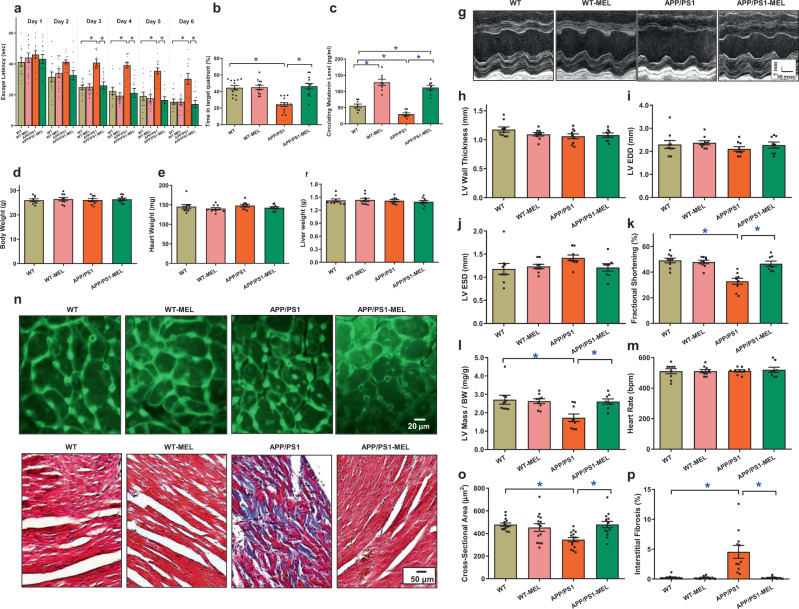
The effect of melatonin (20 mg/kg/day, p.o., 6 weeks) on WT mice and APP/PS1 mice with Alzheimer’s disease. **a** Escape latency; **b** time in target quadrant (%); **c** circulating melatonin level; **d** body weight; **e** hHeart weight; **f** liver weight; **g** representative image of echocardiography; **h** left ventricular (LV) wall thickness; **i** LV end diastolic volume (LVEDD); **j** LV end systolic volume (LVESD); **k** fractional shortening; **l** LV mass/body weight; **m** heart rate; **n** representative images of lectin staining showing cardiomyocyte atrophy (×200) and Masson’s trichrome staining depicting fibrosis (×100); **o** quantitative analysis of cardiomyocyte cross-sectional area; and **p** quantitative analysis of fibrotic area (Masson’s trichrome-stained area in light blue was normalized to total cardiac area). The data are shown as the mean ± SEM, *n* = 7–14 mice per group. *p < 0.05 between the indicated groups

To assess the role of low-circulating melatonin levels in AD-induced cardiac anomalies, mice with APP/PS1 mutations were supplemented with melatonin (20 mg/kg/day, p.o.) for 6 weeks^37^ prior to the assessment of cognitive function, cardiac geometry, and contractile function. The Morris maze test revealed cognitive defects in APP/PS1 mice, as determined by escape latency and time spent in the target quadrant. Although melatonin supplementation itself did not exhibit any effect on cognitive function, it rescued APP/PS1 mutation-induced cognitive defects (Fig. 2a, b). Similar to the human study, circulating melatonin levels were reduced in APP/PS1 mice, the effect of which was masked by melatonin supplementation (Fig. 2c). Neither APP/PS1 mutation nor melatonin supplementation overtly affected body weight or organ weights (Fig. 2d–f and Supplementary Table S2). APP/PS1 mutation induced cardiac geometric and functional anomalies, as evidenced by reduced fractional shortening and normalized LV mass, atrophic remodeling, and interstitial fibrosis. These effects were mitigated by melatonin. Neither APP/PS1 nor melatonin induced any notable changes in LV wall thickness, LV end diastolic diameter, LV end systolic diameter, or heart rate (Fig. 2g–p). These findings indicated a beneficial role for melatonin in AD-induced cognitive and cardiac defects.

### Effect of melatonin on mitochondrial integrity, cardiomyocyte function, and intracellular Ca^2+^ handling

**Fig. 3:**
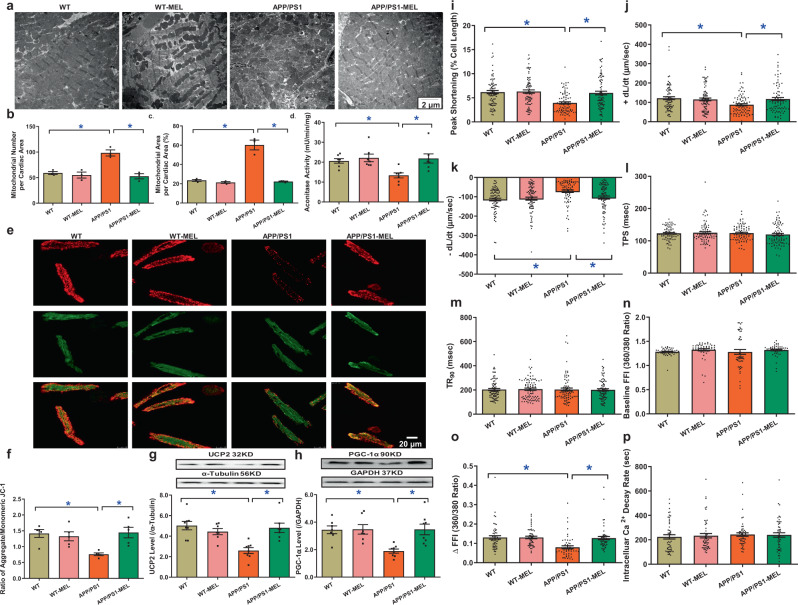
Mitochondrial abnormalities, contractile properties, and intracellular Ca^2+^ homeostasis in cardiomyocytes in WT, APP/PS1 treated with or without melatonin (20 mg/kg/day, p.o., 6 weeks). **a** Representative transmission electron microscopy (TEM) images showing ultrastructural findings; **b** mitochondrial number per cardiac area (×6000); **c** percentage of mitochondrial area per cardiac area (×6000); **d** aconitase activity; **e** representative image of JC-1; **f** ratio of aggregate/monomeric JC-1; **g** UCP2 expression; **h** PGC-1α expression; **i** peak shortening (normalized to cell length); **j** maximal velocity of shortening (+dL/dt); **k** maximal velocity of relengthening (−dL/dt); **l** time-to–peak shortening (TPS); **m** time-to-90% relengthening (TR_90_); **n** resting fura-2 fluorescence intensity (FFI); **o** electrically stimulated rise in FFI (ΔFFI); and **p** single exponential intracellular Ca^2+^ decay. The data are shown as the mean ± SEM, *n* = 3 mice per group for **b** and **c**, *n* = 4–7 mice per group for **d** and **f**, *n* = 6–8 mice per group for **g** and **h**, *n* = 80 cells from 5 mice (∼15–20 cells per mouse) per group for **i**–**m**, and *n* = 50 cells from 5 mice (∼10–15 cells per mouse) per group for **n**–**p**. *p < 0.05 between the indicated groups

To assess the potential mechanisms involved in melatonin-induced benefits, myocardial ultrastructure, mitochondrial integrity and function, and cardiomyocyte mechanical properties were evaluated. The data shown in Fig. 3a–c revealed that the APP/PS1 mutation overtly interrupted myofilament alignment and mitochondrial ultrastructure, as evidenced by the increased numbers and sizes of mitochondria. Although melatonin did not affect myocardial ultrastructure in the controls, it ablated APP/PS1-induced myocardial ultrastructural anomalies. This was supported by mitochondrial assessments. APP/PS1 reduced aconitase activity, the JC-1 ratio, and levels of mitochondrial uncoupling protein 2 (UCP2) and peroxisome proliferator-activated receptor-gamma coactivator 1α (PGC-1α), the effects of which were reversed by melatonin, with little effect of melatonin in the controls (Fig. 3d–h). Assessment of cardiomyocyte mechanics revealed that APP/PS1 mutation overtly impaired cardiomyocyte contractility and intracellular Ca^2+^, as evidenced by decreases in peak shortening (PS), maximal velocity of shortening and relengthening (±dL/dt), and the electrically stimulated rise in intracellular Ca^2+^ (∆FFI) without affecting time-to-PS (TPS), time-to-90% relengthening (TR_90_), resting intracellular Ca^2+^ level, or the intracellular Ca^2+^ decay rate. Consistent with its effect on myocardial contractility, melatonin abrogated APP/PS1-induced defects in cardiomyocyte contractility and intracellular Ca^2+^ without eliciting any effect in the controls (Fig. 3i–p).

### Role of ALDH2 in melatonin-mediated protection against APP/PS1-induced cardiac anomalies

**Fig. 4:**
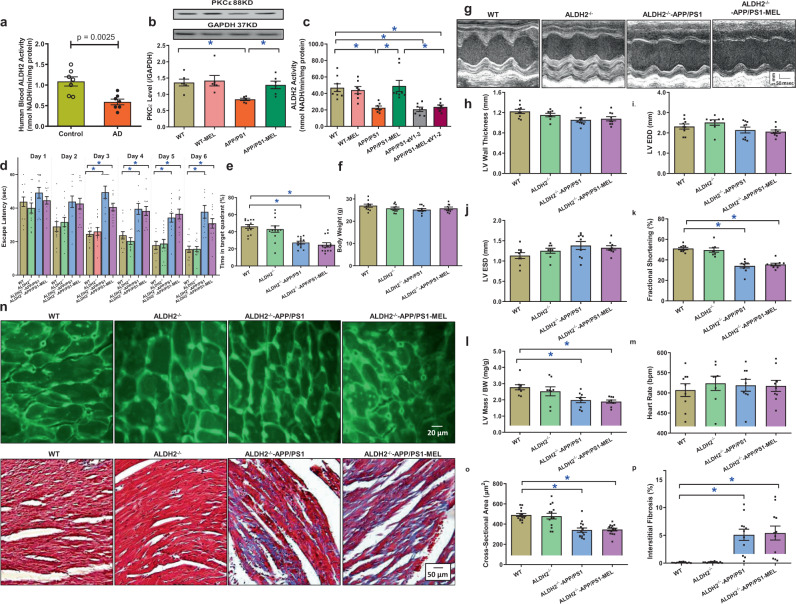
ALDH2 activity and the effect of melatonin (20 mg/kg/day, p.o., 6 weeks) on ALDH2^−/−^-APP/PS1 mice. **a** Blood ALDH2 activity in Alzheimer’s disease patients and age-matched controls. **b** PKCε expression in WT and APP/PS1 mice treated with or without melatonin; **c** cardiomyocyte ALDH2 activity in WT and APP/PS1 mice treated with or without melatonin (100 μΜ) and the PKCε inhibitor εV1–2 (1 µM); **d** escape latency in WT, ALDH2^−/−^, ALDH2^−/−^-APP/PS1 mice, and ALDH2^−/−^-APP/PS1 mice treated with melatonin; **e** time in target quadrant; **f** body weight; **g** representative image of echocardiography; **h** left ventricular (LV) wall thickness; **i** LV end diastolic volume (LVEDD); **j** LV end systolic volume (LVESD); **k** fractional shortening; **l** LV mass/body weight; **m** heart rate; **n** representative images of lectin staining showing cardiomyocyte atrophy (×200) and Masson’s trichrome staining depicting fibrosis (×100); **o** quantitative analysis of cardiomyocyte cross-sectional area; and **p** quantitative analysis of fibrotic area. The data are shown as the mean ± SEM, *n* = 5–7 per group for **a**–**c**, *n* = 9–14 mice per group for **d**–**p**. *p < 0.05 between the indicated groups

Given that a deficiency in the mitochondrial protein ALDH2 was shown to promote the risk of AD,^23,38^ ALDH2 activity was evaluated in the blood from AD patients and cardiac tissues from APP/PS1 mice. Our results revealed reduced ALDH2 activity in blood from AD patients (Fig. 4a). Next, we assessed the levels of the common ALDH2-activating protein kinase Cε (PKCε)^39^ and noted a downregulation in PKCε in the myocardium in APP/PS1 mice, the effect of which was alleviated by melatonin (Fig. 4b). To discern the possible role of PKCε in melatonin-induced regulation of ALDH2 activity, cardiomyocytes from wild-type (WT) and APP/PS1 mice were incubated with melatonin (100 μΜ for 48 h)^37^ in the absence or presence of the specific PKCε inhibitor εV1‑2 (1 μM).^40^ Our data revealed low ALDH2 enzymatic activity but no changes in protein levels in cardiomyocytes from APP/PS1 mice, the effect of which was abrogated by melatonin. Interestingly, pretreatment with the specific PKCε inhibitor εV1‑2 nullified the melatonin-mediated effect on ALDH2 activity without eliciting the effect in the controls (Fig. 4c and Supplementary Fig. S1), indicating that melatonin restored ALDH2 activity in a PKCε-dependent manner. We then repeated the melatonin supplementation study in APP/PS1 mice crossed with ALDH2-knockout mice. The mice were back-crossed several generations to achieve stable APP/PS1 mutation with ALDH2 knockout. ALDH2 knockout failed to elicit any morphological or functional (including cognitive) effects nor did it alter APP/PS1 mutation-elicited changes in cognition, cardiac morphology, and function. Interestingly, melatonin supplementation no longer alleviated APP/PS1 mutation-induced anomalies in cognition, cardiac remodeling, or echocardiographic parameters (Fig. 4d, e, g–p). Neither melatonin nor ALDH2 knockout affected biometric or blood glucose parameters in WT or APP/PS2 mice (Fig. 4f and Supplementary Table S2). These data suggest an obligatory role for ALDH2 in melatonin-mediated benefits against cardiac geometric and functional anomalies in APP/PS1 mutant mice.

### Role of ALDH2 in melatonin-mediated protection against APP/PS1-induced ultrastructural, mitochondrial, cardiomyocyte contractile, and intracellular Ca^2+^ anomalies

**Fig. 5:**
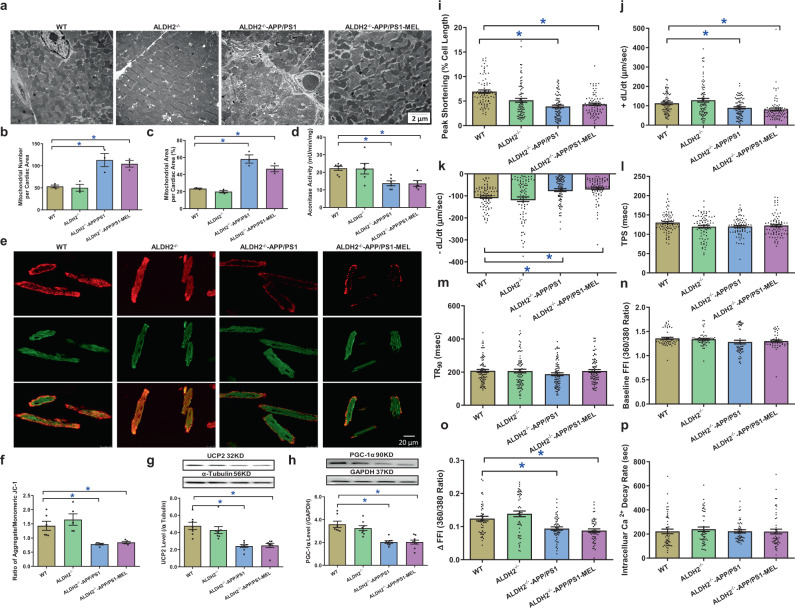
Mitochondrial abnormalities, contractile properties, and intracellular Ca^2+^ homeostasis of cardiomyocytes in WT, ALDH2^−/−^, ALDH2^−/−^-APP/PS1 mice, and ALDH2^−/−^-APP/PS1 mice treated with melatonin (20 mg/kg/day, p.o., 6 weeks). **a** Representative transmission electron microscopy (TEM) images showing the ultrastructural findings; **b** mitochondrial number per cardiac area (×6000); **c** percentage of mitochondrial area per cardiac area (×6000); **d** aconitase activity; **e** representative image of JC-1; **f** ratio of aggregate/monomeric JC-1; **g** UCP2 expression; **h** PGC-1α expression; **i** peak shortening (normalized to cell length); **j** maximal velocity of shortening (+dL/dt); **k** maximal velocity of relengthening (−dL/dt); **l** Time-to–peak shortening (TPS); **m** time-to-90% relengthening (TR_90_); **n** resting fura-2 fluorescence intensity (FFI); **o** electrically stimulated rise in FFI (ΔFFI); and **p** single exponential intracellular Ca^2+^ decay. The data are shown as the mean ± SEM, *n* = 3 mice per group for **b**, **c**, *n* = 4–7 mice per group for **d**–**f**, *n* = 6–8 mice per group for **g**, **h**, *n* = 80 cells from 5 mice (∼15–20 cells per mouse) per group for **i**–**m**, and *n* = 50 cells from 5 mice (∼10–15 cells per mouse) per group for **n**–**p**. *p < 0.05 between the indicated groups

We re-examined morphological and functional parameters in APP/PS1-ALDH2 double mutation-knockout mice supplemented with melatonin. Transmission electron microscopy examination revealed that ALDH2 knockout did not generate any ultrastructural effects, nor did it alter APP/PS1 mutation-induced myofilament alignment and mitochondrial ultrastructural derangement (manifested by increased mitochondrial number and area). Consistent with its effect on cardiac function, ALDH2 knockout mitigated the melatonin-mediated protection against APP/PS1-induced ultrastructural derangement (Fig. 5a–c). This finding was further validated by assessment of mitochondrial function using aconitase activity, JC-1 staining, and the levels of UCP2 and PGC-1α (Fig. 5d–h). Evaluation of cardiomyocyte mechanics supported the obligatory role of ALDH2 in melatonin-elicited preservation of cardiomyocyte contractility and intracellular Ca^2+^ properties in APP/PS1 mice, as evidenced by PS ± dL/dt and ∆FFI, in which melatonin failed to induce any beneficial responses (Fig. 5i–p). These data again support an obligatory role for ALDH2 in melatonin-induced protection on cardiac ultrastructure and mitochondrial integrity/function in APP/PS1 AD mice.

### Role of melatonin membrane receptors in melatonin-mediated protection against APP/PS1-induced cardiomyocyte contractile anomalies

To discern the possible role of melatonin membrane receptors, APP/PS1, ALDH2 knockout, and APP/PS1-ALDH2 double mutation-knockout mice were supplemented with or without melatonin, and the melatonin membrane receptors were evaluated in myocardial tissues. Our results failed to note an overt change in either MT1 or MT2 in WT, APP/PS1, ALDH2-knockout or APP/PS1-ALDH2 mutation-knockout mice with or without melatonin supplementation (Supplementary Fig. S2a, b). Moreover, cardiomyocyte contractile function was re-examined in WT and APP/PS1 mice with or without melatonin treatment (100 μΜ for 4 h)^37^ in the presence or absence of the nonselective melatonin membrane receptor inhibitor luzindole (10 μM).^18^ Our data revealed that luzindole effectively nullified melatonin-mediated cardioprotection of PS and ±dL/dt against APP/PS1 mutation without eliciting any effect on the controls (Supplementary Fig. S2c–h). These data support an obligatory role for melatonin membrane receptors in melatonin-induced cardioprotection in the context of APP/PS1 pathology.

### Effect of melatonin and ALDH2 on APP/PS1-induced changes in apoptosis and inflammation

**Fig. 6:**
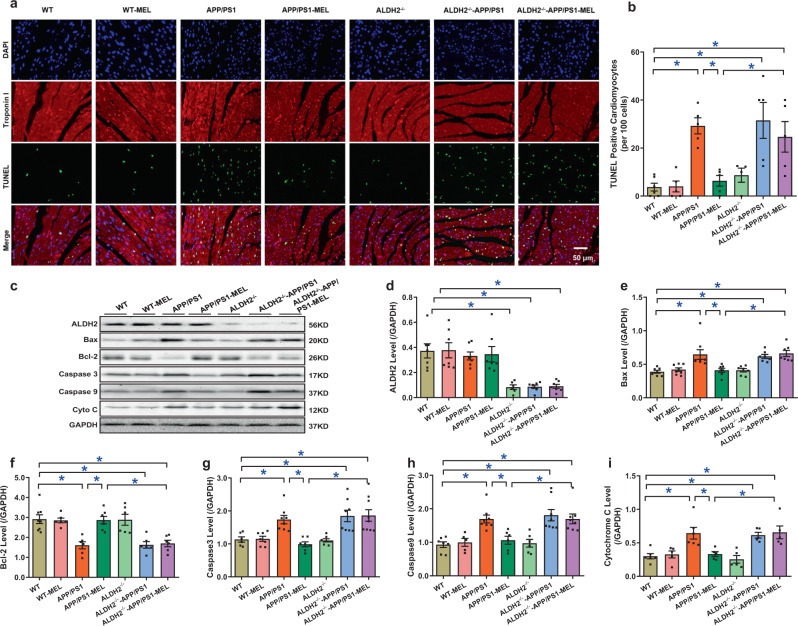
Effect of melatonin on apoptosis levels in WT, APP/PS1, ALDH2-knockout (ALDH2^−/−^), and ALDH2^−/−^-APP/PS1 mice. **a** Representative image of TUNEL; **b** TUNEL-positive cardiomyocytes per 100 cells; **c** representative gel depicting ALDH2, the apoptosis markers Bax, Bcl-2, Caspase 3, Caspase 9, and cytochrome C and GAPDH (loading control) levels using specific antibodies; **d** ALDH2 expression; **e** Bax level; **f** Bcl-2 level; **g** Caspase 3 level; **h** Caspase 9 level; and **i** cytochrome C level. The data are shown as the mean ± SEM, *n* = 5–8 mice per group. *p < 0.05 between the indicated groups

Terminal deoxynucleotidyl transferase dUTP nick end labeling (TUNEL) staining indicated that APP/PS1 mutation overtly promoted apoptosis and the effect was negated by melatonin (Fig. 6a, b). Next, western blot analysis was used to evaluate the levels of apoptotic and proinflammatory protein markers, including Bax, Bcl-2, caspase 3, caspase 9, cytosolic cytochrome C (cyto C), tumor necrosis factor (TNF)-α, and interleukin (IL)-1β. Consistent with the TUNEL staining data, APP/PS1 overtly upregulated the levels of the proapoptotic markers Bax, caspase 3, caspase 9, and cytosolic cyto C, as well as the proinflammatory markers TNF-α and IL-1β, while suppressing the levels of Bcl-2. These effects were abrogated by melatonin, with little effect on the controls. Although ALDH2 knockout failed to elicit any effects on basal or APP/PS1-induced apoptosis and inflammation, it nullified melatonin-mediated beneficial apoptotic and inflammatory responses in APP/PS1 mouse hearts. Neither APP/PS1 mutation nor melatonin supplementation altered ALDH2 expression. ALDH2 ablation was confirmed using western blot analysis (Fig. 6c–i and Supplementary Fig. S3).

### Changes in circulating autophagy markers in AD patients and the effect of melatonin and ALDH2 knockout on APP/PS1-induced changes in cytosolic mtDNA, autophagy, and mitophagy

**Fig. 7:**
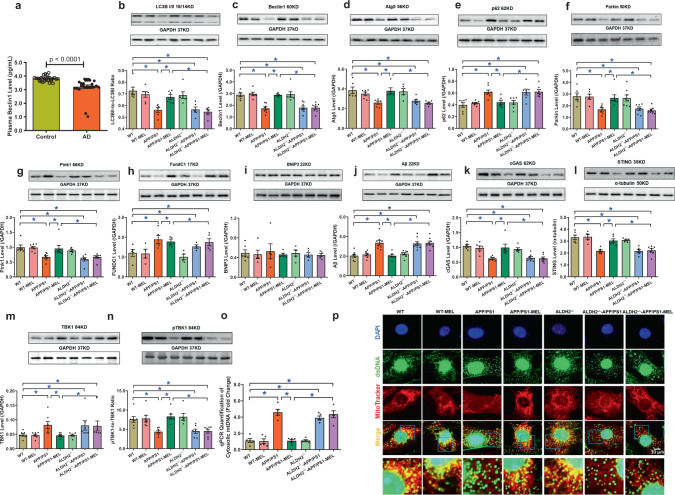
Circulating Beclin1 levels in Alzheimer’s disease patients (*n* = 28) and age-matched controls (*n* = 25), and the effect of melatonin (20 mg/kg/day, p.o., 6 weeks) on autophagy and mitophagy levels in WT, APP/PS1, ALDH2-knockout (ALDH2^−/−^), and ALDH2^−/−^-APP/PS1 mice. **a** Circulating Beclin1 level; **b** LC3BII to I ratio; **c** Beclin1 level; **d** Atg5 level; **e** p62 level; **f** Parkin level; **g** Pink1 level; **h** FundC1 level; **i** Bnip3 level; **j** Aβ level; **k** cGAS level; **l** STING level; **m** TBK1 level; **n** phosphor-TBK1 level; **o** qPCR assessment of in vivo cytosolic mtDNA levels from adult mouse hearts; and **p** representative immunocytochemistry images of cytosolic DNA accumulation in neonatal mouse cardiomyocytes from WT, APP/PS1, ALDH2 knockout (ALDH2^−/−^), and ALDH2^−/−^-APP/PS1 neonates treated with or without melatonin (Mel, 100 μΜ) for 4 h prior to assessing mtDNA using a dsDNA antibody and MitoTracker. mtDNA (dsDNA colocalized with MitoTracker, yellow dots), nuclear DNA (dsDNA in the nucleus colocalized with DAPI), and cytosolic DNA (dsDNA not colocalized with mitochondria or the nucleus, green dots) are presented. APP/PS1 mutation promotes buildup of cytosolic dsDNA, which was mitigated by melatonin treatment, whereas ALDH2 ablation abrogated the beneficial effect of melatonin, *n* = 4 biological repeats for **p**. The data are shown as the mean ± SEM, *n* = 5–9 mice per group. *p < 0.05 between the indicated groups

Circulating levels of Beclin1 were markedly suppressed in AD patients (Fig. 7a). Western blot analysis revealed downregulation of LC3B (LC3BII-to-LC3BI ratio), Beclin1, and Atg5, as well as the mitophagy markers Parkin and Pink1, along with upregulated levels of p62 and the mitophagy marker FundC1, with little change in BNIP3 in the myocardium in APP/PS1 mice, and these effects were negated by melatonin (Fig. 7b–i). Consistent with the change in autophagy/mitophagy, which serves as the essential machinery to engulf and degrade the APP/PS1 product Aβ,^41,42^ the elevated levels of Aβ were drastically decreased by melatonin treatment (Fig. 7j). Consistent with earlier observations, ALDH2 knockout mitigated melatonin-induced beneficial effects on the levels of autophagy, mitophagy, and Aβ in APP/PS1 mice, with little effect on basal or APP/PS1-induced autophagy, mitophagy, or Aβ (Fig. 7b–j).

To ascertain an obligatory role of autophagy in melatonin-mediated cardioprotection, cardiomyocyte contractile function was re-examined in WT and APP/PS1 mice with or without melatonin treatment (100 μΜ for 4 h)^37^ in the presence or absence of the autophagy inhibitor 3-methyladenine (3-MA, 10 mM).^31^ We found that 3-MA abrogated melatonin-mediated cardioprotection of PS and ±dL/dt without inducing any effect on the controls (Supplementary Fig. S2c–h).

To discern the possible signaling mechanisms involved in melatonin- and ALDH2-induced changes in autophagy and mitophagy, cytosolic accumulation of mitochondrial injury-sensitive mtDNA and cGAS-STING signaling were evaluated using qPCR, immunocytochemistry and western blotting. We found that APP/PS1 mutation promoted mtDNA accumulation in the cytosol and downregulated the levels of the DNA damage sensor cGAS and its downstream signaling molecule STING, the effects of which were restored by melatonin supplementation with little effect of melatonin on the controls (Fig. 7k–l, o). As TBK1 serves as a downstream target for cGAS-STING,^43^ we examined total and phosphorylated TBK1. Our data showed that APP/PS1 substantially upregulated levels of total TBK1 while decreasing levels of phosphorylated TBK1 and these effects were abrogated by melatonin, with little effect on the controls (Fig. 7m, n). Although ALDH2 ablation itself did not alter mtDNA and cGAS-STING-TBK1 signaling at either basal conditions or in the context of APP/PS1 mutation, it abrogated the melatonin-mediated changes in cytosolic accumulation of mtDNA or cGAS-STING-TBK1 signaling in the APP/PS1 murine model of AD (Fig. 7k–o). These findings were recapitulated using immunocytochemistry in neonatal mouse cardiomyocytes, in which melatonin rescued APP/PS1 mutation-induced mtDNA buildup in the cytosol, and these effects were abrogated by ALDH2 deletion (Fig. 7p). To evaluate the possible interaction between ALDH2 and cGAS, coimmunoprecipitation was utilized to discern the direct interaction between these proteins. As shown in Supplementary Fig. S4, we failed to identify any interaction between ALDH2 and cGAS.

### Role of cGAS-STING-TBK1 signaling in melatonin- and ALDH2-induced cardiac mitophagy and contractile responses in APP/PS1 experimental AD

**Fig. 8:**
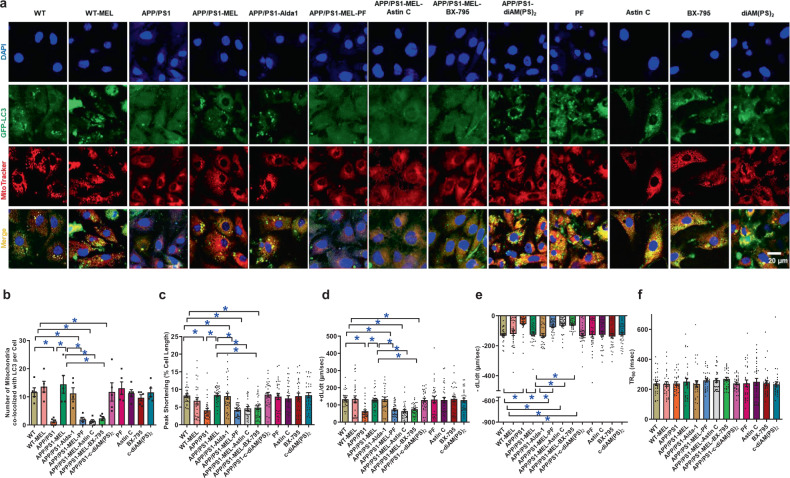
Melatonin restores mitophagy and contractile function through the ALDH2-cGAS-STING-TBK1 signaling pathway. **a** Representative images of GFP-LC3, MitoTracker, DAPI staining, and merged images in WT and APP/PS1 neonatal cardiomyocytes treated with or without melatonin (Mel, 100 μΜ), PF-06928215 (cGAS inhibitor, 10 μM), Astin (STING inhibitor, 10 μM), c-diAM(PS)_2_ (STING activator, 2.7 μM), and BX795 (TBK activator, 1 μM). **b** Quantitative analysis of GFP-LC3 and MitoTracker colocalized puncta per cell; **c** peak shortening (normalized to cell length); **d** maximal velocity of shortening (+dL/dt); **e** maximal velocity of relengthening (−dL/dt); and **f** time-to-90% relengthening (TR_90_). The data are shown as the mean ± SEM, *n* = 5 replicates per group for **b**, *n* = 27–31 cells from 3 mice (∼10 cells per mouse) per group for **c**–**f**. *p < 0.05 between the indicated groups

To discern the cause–effect relationship of cGAS-STING-TBK1 signaling in melatonin- and ALDH2-induced changes in mitophagy, neonatal cardiomyocytes from WT and APP/PS1 mice were transfected with GFP-LC3 prior to treatment with melatonin (100 μM)^37^ for 48 h. Cells was incubated in the presence or absence of melatonin, Alda-1 (20 μM),^21^ the cGAS inhibitor PF-06928215 (10 μM),^44^ the STING inhibitor Astin (10 nM),^45^ the STING activator c-diAM(PS)_2_ (2.7 μM),^46^ and the TBK1 inhibitor BX795 (1 μM).^47^ Consistent with earlier observations, neonatal cardiomyocytes from APP/PS1 mice displayed low levels of mitophagy and this effect was attenuated by melatonin and the ALDH2 activator Alda-1. Although PF-06928215, Astin C, and BX795 failed to exert any effect on mitophagy (GFP-LC3-MitoTracker puncta), these inhibitors abrogated the beneficial effects of melatonin-mediated mitophagy promotion against APP/PS1. Moreover, activation of STING using c-diAM(PS)_2_ mitigated the APP/PS1-induced loss of mitophagy in a manner similar to that of melatonin and Alda-1 (Fig. 8a, b). To further validate the obligatory role of cGAS-STING signaling in melatonin-mediated cardiac benefits, neonatal cardiomyocytes from WT and APP/PS1 mice were transfected with small interfering RNA (siRNA) against cGAS or STING prior to treatment with melatonin (100 μM)^37^ for 48 h. As shown in Supplementary Fig. S5, our data revealed that siRNA against either cGAS or STING canceled the protective effect of melatonin on mitophagy and caspase-3-mediated apoptosis in response to APP/PS1 mutation with little effect on the controls, in a manner similar to that of the pharmacological inhibitors PF-06928215 or Astin. Scramble siRNA had no effect on mitophagy or caspase-3 activity (data not shown).

Adult murine cardiomyocytes from WT and APP/PS1 mice were exposed to melatonin (100 μM)^37^ for 4 h in the absence or presence of Alda-1 (20 μM),^21^ PF-06928215 (10 μM),^44^ Astin (10 nM),^45^ c-diAM(PS)_2_ (2.7 μM),^46^ or the TBK1 inhibitor BX795 (1 μM).^47^ As expected, cardiomyocytes from APP/PS1 mice had overtly compromised cardiomyocyte shortening, as manifested by PS and ±dL/dt, without affecting TPS or TR_90_. These changes were nullified by melatonin, with little effect on the controls. Interestingly, melatonin-mediated benefits were abrogated by PF-06928215, Astin C and BX795, with little effect of these inhibitors on the controls. Activation of STING using c-diAM(PS)_2_ nullified APP/PS1-induced cardiomyocyte contractile defects in a manner similar to that of melatonin and Alda-1, indicating a role for cGAS-STING-TBK1 in APP/PS1- and melatonin-induced changes in cardiomyocyte contractility (Fig. 8c–f).

## Discussion

**Fig. 9:**
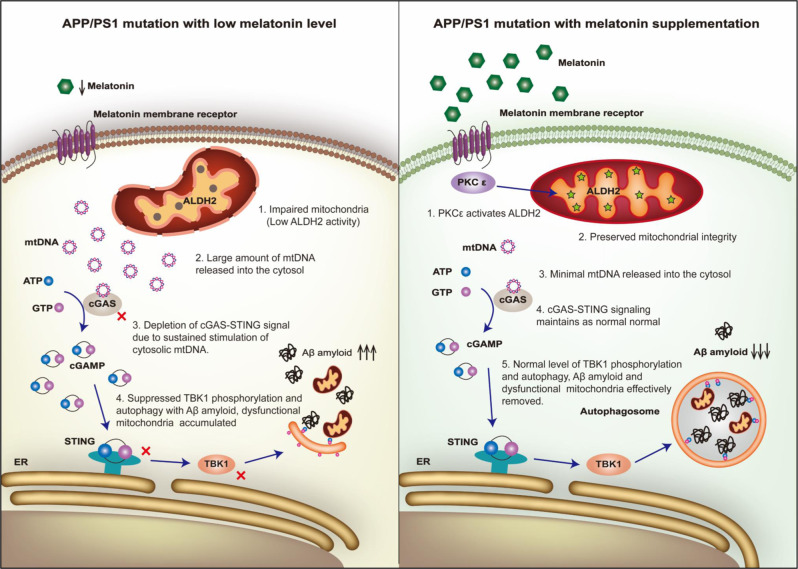
Melatonin protects against AD-associated heart dysfunction through ALDH2 and cGAS-STING-TBK1 signaling. AD is associated with decreased melatonin levels, which leads to suppressed ALDH2 activity. Impaired activity of the mitochondrial protein ALDH2 results in damage to mitochondrial integrity, causing mtDNA to be released into the cytosol. A large amount of mtDNA depletes cGAS-STING-TBK1 signaling, leading to suppressed levels of autophagy and mitophagy. These responses further impair clearance of Aβ and mitochondria, ultimately resulting in heart dysfunction. Melatonin supplementation protects heart function in AD by boosting ALDH2 activity through PKCε, thus restoring mitochondrial integrity, cGAS-STING-TBK1 signaling, and autophagy and mitophagy levels

Our findings suggest that AD patients exhibit cardiac dysfunction in association with low levels of circulating melatonin, Beclin1, and ALDH2 activity. In the present study, melatonin rescued APP/PS1 AD model-induced cardiac remodeling, and dysfunction and defects in mitochondria and autophagy. Melatonin is synthesized in pineal glands and is tightly regulated by the suprachiasmatic nucleus through sympathetic regulation.^35^ Although exactly how AD pathology leads to low-circulating melatonin levels is beyond the scope of our present study, but interrupted circadian cycle (defects in the retina-suprachiasmatic nucleus-pineal pathway),^48^ depletion of the melatonin precursor (serotonin),^49^ and pineal gland calcification and shrinkage^50^ are all known to contribute to AD-induced loss of melatonin. A reduction in circulating melatonin levels was also reported in many other myopathies, including hypertensive cardiomyopathy,^51^ dilated cardiomyopathy,^52^ and LV remodeling following myocardial infarction.^53^ Perhaps, the most intriguing finding from our present study is the involvement of ALDH2-cGAS-STING-TBK1 in the regulation of mitophagy in melatonin-mediated cardioprotection in AD (Fig. 9). Melatonin supplementation ameliorated AD-induced cardiac remodeling and contractile anomalies possibly via restoration of PKCε-ALDH2-cGAS-STING-TBK1-mediated mitophagy. Collectively, these clinical and experimental data revealed not only a novel mechanism for defective mitophagy in AD-induced myocardial pathology but also the therapeutic potential of melatonin in AD-induced cardiac anomalies through ALDH2-mediated maintenance of mitophagy.

Although the impact of neurodegenerative diseases on cardiac homeostasis remains debatable,^1,3^ data from our study suggest overt cardiac remodeling (atrophy) and contractile dysfunction in our APP/PS1 mutant model of AD, which is consistent with clinical observations.^1,3,5^ Similar to previous reports from human^4^ and experimental models,^13,54,55^ our present data demonstrated cardiac dysfunction in AD patients and APP/PS1 mice. To date, membrane (MT1 and MT2) and nuclear (retinoid Z receptor/retinoid-related receptor) receptors, as well as receptor-independent modalities, have been identified for melatonin.^35^ Although few differences were identified in MT1 and MT2 receptors between WT and APP/PS1 mouse hearts, the nonselective melatonin membrane receptor blocker luzindole nullified melatonin-mediated cardioprotection against APP/PS1 mutant AD pathology, indicating a membrane receptor-dependent mechanism for melatonin. Our study noted a beneficial role for melatonin in improving cognitive function in APP/PS1 AD mice. This finding is consistent with previous reports in which melatonin treatment improves cognitive function and hippocampal mitochondrial function, the effects of which seem to be mediated via the MT2 membrane receptor.^56^ In particular, melatonin rescues brain mitochondrial function in APP/PS1 mice with a near complete recovery of mitochondrial respiration, membrane potential, and ATP in the hippocampus, cortex, and striatum.^57^ However, there is controversy regarding changes in melatonin receptors in AD pathology, including both increased and decreased melatonin receptor levels in AD.^56,58^ Our data also revealed that the autophagy inhibitor 3-MA abrogated melatonin-mediated cardioprotection (Supplementaryl Fig. S2), indicating a role for dysregulated autophagy, particularly mitophagy, in AD-induced cardiac defects. Melatonin nullified AD-induced cardiac remodeling, defects in contractility, intracellular Ca^2+^, apoptosis, and mitochondria and mtDNA accumulation. Interestingly, melatonin supplementation also restored the AD-induced loss of autophagy and mitophagy, supporting a pivotal role for autophagy and mitochondrial integrity in melatonin-mediated benefits against AD-induced cardiac defects. These findings were supported by observation of reduced circulating autophagy levels in AD patients, indicating a permissive role for autophagy in AD- and melatonin-induced changes in cardiac geometry and function.

Perhaps, the most intriguing finding from our study is the obligatory role for ALDH2 in melatonin-mediated beneficial effects. In the absence of ALDH2, melatonin-mediated protection against APP/PS1 myopathic changes were abrogated. In our study, crossing APP/PS1 mice with ALDH2-knockout mice did not worsen cognitive deficits or cardiac anomalies, indicating a minimal role of intrinsic ALDH2 loss in AD-induced cognitive and cardiac deficits. To date, the precise role of ALDH2 in the etiology of AD remains debatable. Earlier findings from our group and others indicated a beneficial role for ALDH2 in cardiovascular diseases, including diabetes mellitus, ischemia reperfusion injury, atherosclerosis, stroke, sepsis, and obesity, through regulation of autophagy.^21,30–32,59,60^ In the context of neurodegenerative diseases, ALDH2 deficiency was reported to be correlated with AD incidence,^23,25^ although contradictory findings were noted, disproving the correlation between ALDH2 and AD risk in humans.^28,34,61^ The possible role for ALDH2 in AD pathology is consistent with low ALDH2 activities in AD patients and APP/PS1 mice. Our results further suggested that melatonin facilitated ALDH2 activity through upregulated levels of PKCε, a well-known upstream stimulator of ALDH2.^39^ Inhibition of PKCε abrogated melatonin-induced restoration of cardiac ALDH2 activity in APP/PS1 mice, consistent with the idea that melatonin directly activates PKCε.^62^

Here, our results showed an essential role for the DNA sensor cGAS and its downstream effector STING in APP/PS1 mutation- and melatonin-induced cardiac responses. Melatonin effectively reversed APP/PS1 mutation-induced changes in the levels of mtDNA, cGAS and STING signaling. Both pharmacological and siRNA knockdown of cGAS-STING abrogated melatonin-mediated beneficial effects on mitophagy, cell survival, and cardiac function, indicating an obligatory role of cGAS-STING signaling in melatonin-mediated protection. Recent evidence has established an essential role for cGAS and STING in the regulation of cardiovascular homeostasis.^63,64^ DNA is usually located in the nucleus and mitochondria. Cytosolic accumulation of mtDNA, which is believed to be a result of impaired mitochondrial integrity, as seen in our APP/PS1 murine hearts, triggers the production of the second messenger cGAMP, which then binds and activates the adaptor protein STING to recruit TBK1 and proinflammatory cytokines to disrupt ATP supply and mitochondrial bioenergetics, leading to irreversible cell death.^36,64^ Interestingly, ALDH2 ablation abrogated melatonin-induced protection against mtDNA cytosolic accumulation, as evidenced by qPCR and immunocytochemistry. Given the role of ALDH2 as a mitochondrial chaperone to preserve mitochondrial integrity,^39^ loss of ALDH2 in the APP/PS1 AD model likely results in mtDNA damage and thus cytosolic mtDNA accumulation, which is restored by melatonin-mediated recruitment of ALDH2 to preserve mitochondrial integrity (as evidenced by mitochondrial function and ultrastructure). Notably, ALDH2 knockout itself did not affect mtDNA or cGAS-STING levels (nor were there cardiac or cognitive phenotypes), indicating that ALDH2 deficiency itself may not be innately harmful in the absence of pathological stress.

Surprisingly, our data showed overtly suppressed cGAS-STING signaling in the presence of profound cytosolic mtDNA accumulation and proinflammatory cytokine release in APP/PS1 murine hearts. This seems to be contrary to mtDNA-induced activation of cGAS-STING signaling. Multiple scenarios may be considered. (1) Sustained stimulation of cGAS by mtDNA buildup, such as in our APP/PS1 model, likely results in depletion of cGAS-STING stores, although the endpoint of the proinflammatory response still prevails. The presence of prolonged and massive cytosolic mtDNA levels may exhaust cGAS-STING signaling and result in decreased cGAS-STING, as noted in our study. Melatonin, through PKC, activates ALDH2 and restores mitochondrial integrity to prohibit mtDNA leakage into the cytosol. Therefore, melatonin may normalize cGAS-STING signaling by eliminating excessive cytosolic mtDNA stimulation of cGAS-STING (Fig. 9). (2) Although it is beyond the scope of our current study, cGAS-STING signaling may be distinct in various cell types. Currently, the cGAS-STING signaling cascade is most well studied in immune cells with limited information in other tissues. Cardiomyocytes are not commonly exposed to pathogenic insults and remain inactive in the context of pathogen defense, similar to macrophages and other immune cells. Thus, cardiomyocytes may exhibit a disparate cGAS-STING signaling response upon mtDNA buildup.

TBK1, an effector of cGAS-STING signaling,^36^ plays a role in the induction of autophagy and mitophagy, possibly through suppression of mammalian target of rapamycin, and recruitment and phosphorylation of p62, as well as by promoting assembly of ubiquitin chains on mitochondria.^65,66^ Our data suggest that TBK1 phosphorylation was concomitantly reduced with mitophagy in APP/PS1 mice regardless of the presence or absence of ALDH2 knockout. Our data further revealed that melatonin restored both mitophagy (Fundc1, likely due to a compensatory response) and TBK1 activity. It is conceivable that the beneficial effect of melatonin is mediated through its regulation of TBK1. Indeed, with inhibition of TBK1, melatonin-mediated preservation of mitophagy was nullified, suggesting an indispensable role of TBK1 in melatonin-mediated protection against AD-associated myopathic changes. More recent evidence indicated a more variable role for cGAS-STING-TBK1 beyond immunity, including metabolic derangement.^36,67^ Notably, STING may directly activate LC3 to promote autophagy independent of TBK1.^68,69^ However, our data indicate an indispensable role of TBK1 in mitophagy induction and cardiomyocyte homeostasis. The vital role of the cGAS-STING-TBK1 axis in melatonin-induced protection against the APP/PS1 mutation was validated with RNA interference or pharmacological modulators of the cGAS-STING-TBK1 signal cascade. It has been hypothesized that autophagy is ultimately responsible for autophagic degradation of Aβ, thus alleviating AD pathology.^41,42^ This is consistent with the result showing that 3-MA ablated melatonin-mediated benefits and changes in Aβ levels in our study (Fig. 9).

The present study raised a number of additional questions. First, it appears that melatonin membrane and nuclear receptors facilitate disparate signaling cascades in different organs, such as hearts and brains. The specific role of melatonin receptors (and receptor-independent mechanisms) in the regulation of cognitive function deserves further investigation. Second, a number of pharmacological inhibitors and activators were used in our study to examine the role of the cGAS-STING-TBK1 signaling cascade in AD-induced cardiac anomalies, although the role of cGAS-STING-TBK1 signaling remains elusive in cognitive dysfunction. Little information is readily available on the pharmacodynamics, pharmacokinetics, specificity and efficacy of these reagents in physiological settings. Further study is needed to elucidate the role of the cGAS-STING-TBK1 signaling cascade in neurological function using genetically engineered animal models or pharmacological agents. Finally, determining the impact of cGAS-STING-TBK1 in AD-induced cardiac anomalies in humans will be of interest.

In conclusion, our study provides the first evidence that melatonin reverses APP/PS1 mutation-induced cardiac dysfunction and mitochondrial injury through ALDH2-cGAS-STING-TBK1-mediated regulation of mitophagy. This is supported by the observation that inhibition of cGAS-STING-TBK1 counters melatonin-mediated beneficial responses. More importantly, our clinical data offer a proof-of-concept correlation of blood levels of melatonin, ALDH2 activity, and autophagy with cardiac dysfunction in AD patients. Although it is still premature to consider the clinical values of melatonin and mitophagy in AD-induced myopathic changes, our results shed some light on the utility of melatonin as an alternate mitophagy inducer (few side effects) in cardiac anomalies in neurodegenerative diseases.

## Materials and methods

For full “Materials and Methods”, please refer to the Supplementary Materials and Methods.

### Murine model of Alzheimer’s disease, generation of ALDH2^−/−^-APP/PS1 mice, and melatonin treatment

All animal procedures were approved by the Animal Care and Use Committees at the Xijing Hospital, the Air Force Military University (Xi’an, China) and University of Wyoming (Laramie, WY), and were in compliance with the Guide for the Care and Use of Laboratory Animals published by NIH. In brief, 10-month-old male APPswe/PS1dE9 (APP/PS1) mutant mice were utilized as the AD model. APP/PS1 mice were crossed with ALDH2^−/−^ mice (both on the C57BL/6 background) to generate heterozygotes, which were further crossed to generate ALDH2^−/−^-APP/PS1 mice. Groups of adult WT, APP/PS1, and ALDH2^−/−^-APP/PS1 mice were treated with melatonin (20 mg/kg/day, oral gavage, 6 weeks).^37^

## Supplementary information

STTT-00690_Supplemental_Final

## Data Availability

The datasets used and/or analyzed to support the findings of this study are available in this paper or the Supplementary Information. Any other raw data that support the findings of this study are available from the corresponding author upon reasonable request.
