# Supplementary material for: ALDH2 contributes to melatonin-induced protection against APP/PS1 mutation-prompted cardiac anomalies through cGAS-STING-TBK1-mediated regulation of mitophagy
Source: Signal Transduct Target Ther. 2020 Jul 24;5:119. doi: 10.1038/s41392-020-0171-5 (PMC7378833; doi:10.1038/s41392-020-0171-5)
Supplement: Supplementary file 1 — STTT-00690_Supplemental_Final [file 41392_2020_171_MOESM1_ESM.doc]

**ALDH2 Contributes to Melatonin-Offered Protection against APP/PS1 Mutation-Induced Cardiac Anomalies through cGAS-STING-TBK1-Mediated Regulation of Mitophagy**

(This file contains supplemental methods, supplementary table S1-S2, supplemental figure S1-S5)

**Shuyi Wang^1,2*^, Lin Wang^3*^, Xing Qin^4^, Subat Turdi^1^, Dongdong Sun^4^, Bruce Culver^1^, Russel J. Reiter^5^, Xiaoming Wang^3^, Hao Zhou^1,6^, and Jun Ren^1,3,7^**

**^1^Center for Cardiovascular Research and Alternative Medicine, University of Wyoming College of Health Sciences, Laramie, WY 82071 USA; ^2^Department of Emergency, Shanghai Tenth People’s Hospital, Tongji University School of Medicine, Shanghai, 200072, China; ^3^Department of Geriatrics, and ^4^Department of Cardiology, Xijing Hospital, the Air Force Military Medical University, Xi'an, China 710032; ^5^Department of Cellular and Structural Biology, UT Health San Antonio, TX, USA; ^6^Chinese PLA General Hospital, Medical School of Chinese PLA, Beijing, 100853, China; ^7^Department of Cardiology and Shanghai Institute of Cardiovascular Diseases, Zhongshan Hospital Fudan University, Shanghai, 200032, China**

***Equal contribution**

**Short title: Melatonin, ALDH2, and heart defect in Alzheimer disease,**

**Correspondence to:**

**Dr. Jun Ren (**[**jren@uwyo.edu**](mailto:jren@medicine.nodak.edu)**) or Dr. Hao Zhou (**[**zhouhao301@outlook.com**](mailto:zhouhao301@outlook.com)**) or Dr. Xiao-Ming Wang (**[**xmwang@fmmu.edu.cn**](mailto:xmwang@fmmu.edu.cn)**)**

**SUPPLEMENTAL METHODS**

***Murine model of Alzheimer’s disease and melatonin preparation:*** Characterization of APP/PS1 line was described in detail previously.[^1^](#_ENREF_1) Genotyping of APP/PS1 mice was completed using DNA isolated from tail clips and polymerase chain reaction (PCR) technique. All primers were synthesized by Integrated DNA Technologies Inc. (Coralville, IA, USA) with the following sequences. APPswe: 5'-GAC TGA CCA CTC GAC CAG GTT CTG -3' and 5'- CTT GTA AGT TGG ATT CTC ATA TCC G-3';  PSEN1dE9: 5'-AAT AGA GAA CGG CAG GAG CA-3' and 5'-GCC ATG AGG GCA CTA ATC AT-3').  Melatonin was dissolved in ethanol and then diluted in sterile water with final concentration < 0.5% ethanol.[^2^](#_ENREF_2) Mice were housed in a climate-controlled environment (22.8 ± 2.0^o^C, 45 – 50% humidity) with a 12/12 – light/dark cycle with *ad libitum* access to tap water and regular lab chow.

***Circulating melatonin and Beclin1 analysis:*** For human Beclin1 and melatonin examination, venous blood was collected from AD patients and their age-matched controls (Supplemental Table 1) and was allowed to clot at room temperature for 1 hr. Serum samples were collected and stored at −80 °C until use. Serum levels of beclin-1 and melatonin were measured using the enzyme-linked immunosorbent assay (ELISA) method with a human beclin-1 ELISA kit (Catalog #E-EL-H0564c, Elabscience Biotechnology, Wuhan, China) and a human melatonin ELISA kit (Catalog # DM E-4600, LDN, Germany) according to manufacturer’s protocols.[^3^](#_ENREF_3) [^4^](#_ENREF_4) Levels of beclin-1 and melatonin were expressed as pg of protein/ml of serum. The mean intra-assay and inter-assay coefficients of variation were below 9.6%.

***ALDH2 activity:*** Human ALDH2 activity were measured using whole blood samples. Mouse ALDH2 activity were measured using primary adult cardiomyocytes isolated from WT and APP/PS1 mice (as described below) treated with or without melatonin (100 μΜ)[^2^](#_ENREF_2) and PKCε inhibitor εV1-2 (1 µM)[^5^](#_ENREF_5). ALDH2 activity were measured in 33 mM sodium pyrophosphate containing 0.8 mM NAD^+^, 15 μM propionaldehyde and 0.1 ml protein extract. Propionaldehyde, the substrate of ALDH2, was oxidized in propionic acid, while NAD^+^ was reduced to NADH to estimate ALDH2 activity. NADH was determined by spectrophotometric absorbance at 340 nm. ALDH2 activity was expressed as nmol NADH/min per mg protein.[^6^](#_ENREF_6)

***Morris water maze test:*** Morris water maze test was conducted in a 120-cm (diameter) tank filled with opaque water (25^o^C) surrounded by dark walls containing geometric designs that served as distal visual cues. In the first phase of training, mice were required to find a platform (15x15cm) marked with a colored pole, placed in different quadrants of the pool. Each animal underwent two trials per day for 6 days, with a maximum of 60 s to find the submerged platform. If mice failed to find the platform within 60s, they were physically guided to it and were allowed to remain on the platform for 20s. After visible platform training, two consecutive days of hidden platform training was conducted in which the platform is submerged in the opaque water 1 cm deep, with 2 trials per day. A probe trial was performed at the end of training, in which the platform was removed. Performance in tasks (latency to platform, time spent in target quadrant) was recorded by a computer-based video tracking system.[^7^](#_ENREF_7)

***Assessment of cognitive score and heart function in human subjects:*** Patients with confirmed Alzheimer’s disease were enrolled to Xijing Hospital, Xi’an, China. Cognitive scores were performed using the Mini-mental state examination (MMSE). Aged-matched otherwise healthy individuals from the same suburb were enrolled to serve as controls. All subjects were provided informed consent for participation who underwent echocardiography assessment, and blood specimen collection. This study protocol was approved by the Xijing Hospital ethnics committee. Exclusion criteria for subjects were: (1) subjects with hypertension based on World Health Organization criteria; (2) subjects with serious diseases such as cancer.

***Echocardiographic assessment:*** Cardiac geometry and function were evaluated in anesthetized (ketamine 80 mg/kg and xylazine 12 mg/kg, i.p.) mice using a 2-dimensional (2-D) guided M-mode echocardiography (Vevo 2100, FUJIFILM Visualsonics, Toronto, ON, Canada) equipped with a 22-55 MHz linear transducer (MS550D, FUJIFILM VisualSonics, Toronto, ON, Canada). Hearts were first imaged in the 2-D mode using the parasternal long-axis view (with a depth of 2 cm) before switching to the M-mode positioned perpendicular to interventricular septum and posterior wall of left ventricle (LV). Diastolic wall thickness, left ventricular (LV) end diastolic dimension (EDD) and LV end systolic dimension (ESD) were measured. LV fractional shortening was calculated as [(EDD-ESD)/EDD] × 100. Estimated LV mass was calculated as [(LVEDD + septal wall thickness + posterior wall thickness)^3^-LVEDD^3^]*1.055, where 1.055 (mg/mm^3^) represents the density of myocardium. Heart rate was averaged in 10 cardiac cycles.[^8^](#_ENREF_8)

***Histological examination:*** After anesthesia, hearts were excised and immediately placed in 10% neutral-buffered formalin at room temperature for 24 hrs after a rinse with PBS. The specimens were embedded in paraffin, cut into 5-μm sections and stained with fluorescein isothiocyanate (FITC)-conjugated wheat germ agglutinin. Cardiomyocyte cross-sectional areas were calculated on a digital microscope (× 400) using the Image J (version1.34S) software.[^9^](#_ENREF_9) The Masson’s trichrome staining was used to detect fibrosis in heart sections. The percentage of fibrosis was calculated using the histogram function of the photoshop software. Briefly, 7 random fields (6 mm^2^) at 200x magnification from each section were assessed for fibrosis. The fraction of the light blue stained area normalized to the total area was used as an indicator of fibrosis while omitting fibrosis of the perivascular, epicardial and endocardial areas from the study.[^10^](#_ENREF_10)

***Aconitase activity:*** Aconitase activity assay (Aconitase activity assay kit, Aconitase-340 assay™, OxisResearch, Portland, OR) was performed according to manufacturer’s instructions. Tissues were homogenized in Tris–HCl pH 7.4 and supernatant were collected. Samples were mixed with trisodium citrate, isocitrate dehydrogenase and NAPD and incubated for 15 min at 37 °C. Absorbance was recorded at 340 nm for 5 min at 37°C and aconitase activity were calculated.[^8^](#_ENREF_8)

***Adult cardiomyocyte isolation:*** After ketamine/xylazine sedation, hearts were rapidly removed and were mounted onto a temperature-controlled (37^o^C) Langendorff system. After perfusing with a modified Tyrode solution (Ca^2+^ free) for 2 min, the heart was digested for 16 - 20 min with a Ca^2+^-free KHB buffer containing Liberase Blendzyme 4 (Hoffmann-La Roche Inc., Indianapolis, IN). The modified Tyrode solution (pH 7.4) contained the following (in mM): NaCl 135, KCl 4.0, MgCl_2_ 1.0, HEPES 10, NaH_2_PO_4_ 0.33, glucose 10, butanedione monoxime 10, and the solution was gassed with 5% CO_2_-95% O_2_. The digested heart was then removed from the cannula and the left ventricle was cut into small pieces in the modified Tyrode’s solution. Tissue pieces were gently agitated and the pellet of cells was resuspended. Extracellular Ca^2+^ was added incrementally back to 1.20 mM over a period of 30 min. A yield of at least 60-70% viable rod-shaped cardiomyocytes with clear sarcomere striations was achieved. Only rod-shaped cardiomyocytes with clear edges were selected for mechanical and intracellular Ca^2+^ studies.

***Cell shortening/relengthening:*** Mechanical properties of myocytes were assessed using a SoftEdge Myocam® system (IonOptix Inc., Milton, MA). IonOptix SoftEdge software was used to capture changes of cardiomyocyte length during shortening and re-lengthening. In brief, cardiomyocytes were placed in a Warner chamber mounted on the stage of an inverted microscope (Olympus IX-70) and superfused (1 ml/min at 25^o^C) with a buffer containing (in mM): 131 NaCl, 4 KCl, 1 CaCl_2_, 1 MgCl_2_, 10 glucose and 10 HEPES, at pH 7.4. Myocytes were field stimulated with supra-threshold voltage at a frequency of 0.5 Hz, 3 msec duration, using a pair of platinum wires placed on opposite sides of the chamber connected to a FHC stimulator (Brunswick, NE). A cohort of cells was exposed to melatonin (100 μM) [^2^](#_ENREF_2) the cGAS inhibitor PF-06928215 (10 μM),[^12^](#_ENREF_12) the ALDH2 activator Alda-1 (20 μM),[^13^](#_ENREF_13) the STING inhibitor Astin (10 nM),[^14^](#_ENREF_14) the STING activator c-diAM(PS)_2_ (2.7 μM)[^15^](#_ENREF_15), the TBK inhibitor BX795 (1 μM)[^16^](#_ENREF_16), autophagy inhibitor 3-MA (10 mM)[^6^](#_ENREF_6) or melatonin membrane receptor antagonist luzindole (5 μM)[^17^](#_ENREF_17) for 4 hrs. IonOptix SoftEdge software was used to capture changes in cell length during shortening and relengthening. Cell shortening and relengthening were assessed using the following indices: peak shortening (PS) – the amplitude myocytes shortened on electrical stimulation, which is indicative of peak ventricular contractility; time-to-PS (TPS) – the duration of myocyte shortening, which is indicative of contraction duration; time-to-90% relengthening (TR_90_) – the duration to reach 90% re-lengthening, which represents cardiomyocyte relaxation duration (90% rather 100% re-lengthening was used to avoid noisy signal at baseline concentration); and maximal velocities of shortening (+ dL/ dt) and relengthening (− dL/dt) – maximal slope (derivative) of shortening and relengthening phases, which are indicatives of maximal velocities of ventricular pressure rise/fall.[^8^](#_ENREF_8)

***Intracellular Ca^2+^ transients:*** A cohort of myocytes was loaded with fura-2/AM (0.5 (M) for 10 min and fluorescence intensity were recorded with a dual-excitation fluorescence photomultiplier system (Ionoptix, Milton, MA). Cardiomyocytes were placed onto an Olympus IX-70 inverted microscope and imaged through a Fluor x 40 oil objective. Cells were exposed to light emitted by a 75W lamp and passed through either a 360 or a 380 nm filter, while being stimulated at 0.5 Hz. Fluorescence emissions were detected between 480-520 nm and qualitative change in fura-2 fluorescence intensity (FFI) was inferred from FFI ratio at the two wavelengths (360/380). Fluorescence decay time was measured as an indication of intracellular Ca^2+^ clearing rate.[^8^](#_ENREF_8)

***Mitochondrial membrane potential measurement:*** Mitochondrial function was evaluated via detecting the mitochondrial membrane potential. In brief, cells were incubated with the MitoProbe™ JC-1 assay kit (Thermo Fisher Scientific Inc.) for 30 min at 37˚C in the dark. Subsequently, cold-PBS was used to wash cells twice to remove the free JC-1 probe. Then, nuclei were stained with DAPI for 3 min at room temperature and the mitochondrial potential was assessed under an Olympus IX81 inverted microscope (magnification, x200) using FV10-ASW 1.7 software (both from Olympus Corporation, Tokyo, Japan). Fluorescence images (red and green fluorescence) were first converted to the grayscale pictures with the help of Image-Pro Plus 4.5 software (NIH, Bethesda, MD, USA). Then, red/green fluorescence intensities were separately recorded as the grayscale intensity. Subsequently, relative grayscale intensity was expressed as a ratio to that of control group. Mitochondrial potential was quantified via evaluating the ratio of red-to-green fluorescence intensity.[^18^](#_ENREF_18)

***Neonatal mouse cardiomyocyte harvest:*** Whole hearts were harvested from neonatal (1–2 day-old) WT and APP/PS1 mice. Hearts were cut into small pieces before digestion with 0.25% trypsin (Carolina, Burlington, NC, USA). Primary neonatal cardiomyocytes were collected as previously described, plated in confocal petri dish and cultured for 48 h at 37 °C in the presence of 95% O_2_ and 5% CO_2_.[^19^](#_ENREF_19)

***Quantification of the GFP-LC3*** ***mitophagy*** ***and RNA interference:***  Co-localization of GFP-LC3 with mitoTracker was used to visualize mitophagy. Neonatal mouse cardiomyocytes from WT and APP/PS1 mice were transfected with GFP-LC3 adenovirus for 48 hrs followed by drug treatment or RNA interference. A cohort of cells was exposed to melatonin (100 μM)[^2^](#_ENREF_2), the cGAS inhibitor PF-06928215 (10 μM),[^12^](#_ENREF_12) the ALDH2 activator Alda-1 (20 μM),[^13^](#_ENREF_13) the STING inhibitor Astin (10 nM),[^14^](#_ENREF_14) the STING activator c-diAM(PS)_2_ (2.7 μM)[^15^](#_ENREF_15), or the TBK inhibitor BX795 (1 μM)[^16^](#_ENREF_16) for 4 hrs. For RNA interference, neonatal cardiomyocytes were transfected with cGAS siRNA or STING siRNA (GenePharma, Shanghai, China) for 72 hrs per the manufacturer’s manual. Each gene was targeted from a pool of 2-3 siRNAs with the most potent one chosen for study. Scrambled siRNA was used in parallel for the control group.[^20^](#_ENREF_20) A cohort of siRNA (against cGAS or STING)-treated neonatal cardiomyocytes was then incubated with melatonin (100 μM)[^2^](#_ENREF_2) for 4 hrs prior to GFP-LC3 mitophagy assessment. Following drug or siRNA treatment, cells were incubated with MitoTracker (500 nM, Cell Signaling Technology, Danvers, MA, USA) for 30 min at 37ºC before fixed with 4% ice-cold paraformaldehyde for 20 min. After 3 rinses with PBS, cells were stained with DAPI for nucleus visualization.  Images were acquired using a Zeiss LSM 710 confocal microscope (Carl Zeiss MicroImaging GmbH, Jena, Germany) at 400× magnification. Data were performed in at least five independent experiments.[^21^](#_ENREF_21)

***TEM:*** Transmission electron microscopy were used to study myocardial ultrastructure. Heart tissues were fixed, trimmed, dehydrated and embedded as previously described.[^19^](#_ENREF_19) Tissue blocks were cut into sections using RMC-MTXL ultramicrotome and a Diatome diamond knife. Images were acquired with a Hitachi 7500 transmission electron microscope.

***TUNEL staining:*** DNA strand breaks were detected using TUNEL staining (In Situ Death Detection Kit, Roche, Branchburg, NJ, USA). In brief, cardiomyocytes were identified using anti-Troponin (1:100, Cell Signaling Technology, Danvers, MA, USA) antibody and nucleus were stained with DAPI. Images were acquired using an Inverted Leica TCS-SP2 AOBS confocal laser-scanning microscope (Leica Camera, Wetzlar, Germany). TUNEL positive cells were counted manually and the ratio of TUNEL positive cells over total nucleus were calculated.[^22^](#_ENREF_22)

***Cytosolic mtDNA assessment:*** Cytosolic mtDNA were assessed following previous reports. In brief, hearts were homogenized in 100 mM Tricine-NAOH solution containing 0.25 M sucrose, 1 mM EDTA and protease inhibitor with a pH of 7.4. Samples were then centrifuged at 4^o^C at 700x g for 10 min to remove nucleus and cellular debris. Supernatant were collected prior to centrifugation at 10,000x g for 30 min at 4^o^C to remove mitochondria. Supernatant were corresponded to cytosolic fraction and were used for DNA isolation using a DNeasy Blood & Tissue Kit (Qiagen, Hilden, Germany). Levels of mtDNA were determined using quantitative PCR (qPCR) using cytochrome c oxidase 1 (mtCOI) with the following primers: forward, 5'-GCCCCAGATATAGCATTCCC-3'; reverse, 5'-GTTCATCCTGTTCCTGCTCC-3'. In addition, cytosolic mtDNA were also evaluated in vitro using immunofluorescence staining in neonatal mouse cardiomyocytes.[^25^](#_ENREF_25) In brief, neonatal cardiomyocytes from WT, APP/PS1, ALDH2^-/-^ and ALDH2^-/-^-APP/PS1 mice were treated with or without melatonin (Mel, 100 μΜ)[^2^](#_ENREF_2) for 4 hrs. Cells were stained with MitoTracker (500 nM, Cell Signaling Technology, Danvers, MA, USA) for 30 min at 37ºC prior to fixation with ice-cold paraformaldehyde for 20 min and permeabilization with 0.5% Triton X-100 for 10 min. Neonatal mouse cardiomyocytes were incubated with PBST containing 1% BSA and 22.5 mg/ml glycine for 1 hr to block the unspecific binding followed by incubation with an anti-dsDNA primary antibody (1:500, Abcam, Cambridge, MA, USA) in a humidified chamber overnight at 4^o^C. Cells were incubated with the Alexa Fluor 488-conjugated secondary antibody (1:500, Abcam, Cambridge, MA, USA) for 1 hr at room temperature prior to DAPI staining. Images were acquired using a Zeiss LSM 710 confocal microscope (Carl Zeiss MicroImaging GmbH, Jena, Germany) at the 630× magnification.

***Caspase-3 assay:*** Neonatal cardiomyocytes from WT and APP/PS1 mice were transfected with siRNA against cGAS or STING for 72 hrs prior to treatment with or without melatonin (Mel, 100 μΜ)[^2^](#_ENREF_2) for 4 hrs. Cell were then collected, centrifuged at 10,000 *g* at 4°C for 10 min, and cell pellets were lysed in an ice-cold cell lysis buffer (50 mM HEPES, 0.1% CHAPS, 1 mM dithiothreitol, 0.1 mM EDTA, 0.1% NP40). Following cell lysis, 60 μl of reaction buffer was added to cell lysate (25 μl), followed by an additional 15 μl of caspase-3 colorimetric substrate (Ac-DEVD-pNA) and was incubated at 37°C for 1 hr, during which time the caspase in the sample was allowed to cleave the chromophore pNA from the substrate molecule. Samples were then read with a microplate reader at 405 nm. Caspase-3 activity was expressed as picomoles of pNA released per μg protein per min.[^8^](#_ENREF_8)

***Immunoprecipitation:*** Co-IP assay was conducted using a commercial Co-IP kit (Pierce, Thermo Fisher Scientific, Waltham, MA, USA). In brief, 50 μg purified ALDH2 antibodies were immobilized with coupling resin. Protein extracts (500 μg) were incubated with antibody-coupled resin gently end-over-end mixing for 2 hrs at room temperature. The resin was washed, and protein complexes bound to the antibody were eluted with 50 μl of elution buffer. The eluted protein was boiled and separated by 10% SDS-PAGE, transferred to a nitrocellulose membrane, and incubated with anti-cGAS antibody. Antibody binding was detected using the enhanced chemiluminescence. The film was scanned and the intensity of immunoblotting bands was detected with a Bio-Rad Calibrated Densitometer (model GS-800).[^13^](#_ENREF_13)

***Western blot analysis:*** Proteins were extracted from heart tissues using the lysis buffer containing 20 mM Tris (pH 7.4), 1 mM EDTA, 1 mM EGTA, 150 mM NaCl,1% Triton, 0.1% sodium dodecyl sulfate, and a protease inhibitor cocktail. Proteins were incubated with anti-UCP2, anti-FundC1,  anti-MT1, anti-MT2, anti-TNF α, anti-IL-1β (Abcam, Cambridge, MA, USA), anti-ALDH2 (Santa Cruz Biotechnology, Dallas, TX , USA), anti-PKCε, anti PGC-1α, anti-Bax, anti-Bcl-2, anti-Caspase 3, anti-Caspase 9, anti-Cychrome C, anti-LC3B, anti-Beclin1, anti-Atg5, anti-p62, anti-Parkin, anti-Pink1, anti-Bnip3, anti-Aβ amyloid, anti-TBK1, anti-phospho-TBK1 (Ser^172^), anti-STING, anti-cGAS (Cell Signaling Technology, Danvers, MA, USA), and anti-glyceraldehyde-3-phosphate dehydrogenase (GAPDH; loading control) (Cell Signaling Technology) antibodies. Horseradish peroxidase-coupled secondary antibodies were used for membrane incubation. After immunoblotting, the films were scanned and detected with a Bio-Rad calibrated densitometer and the intensity of immunoblot bands was normalized to corresponding GAPDH.

***Data analysis*:** Data are presented as Mean ± SEM. Statistical significance (p < 0.05) for each variable was estimated by student’s t-test or one-way analysis of variance followed by Tukey’s test for the *post hoc* analysis.

***Data availability:*** The datasets used and/or analyzed supporting this study are available in this paper, the supplemental data or from the corresponding authors upon reasonable request.

Supplemental Data Table S1: General anthropometric features of control and AD patients

|  | Control (26) | AD (26) |
| --- | --- | --- |
| Age (yrs) | 80.7 ± 1.4 | 84.5 ± 1.5 |
| MMSE | 26.6 ± 0.4 | 10.0 ± 0.9* |
| Male | (11/26) 42% | (15/26) 58% |
| Female | (15/26) 58% | (11/26) 42% |
| Type 2 diabetes | 2/26 | 2/26 |
| Cerebral infarction | 1/26 | 2/26 |

MMSE: Mini-mental state examination, values lower than 24 denote dementia, Patient size is given in parenthesis. Data are shown in Mean ± SEM, *p *<* 0.05 *vs*. Control group.

Supplemental Data Table S2: General characteristics of WT and APP/PS1 mice with or without melatonin treatment

| Parameter | WT | WT-Melatonin | APP/PS1 | APP/PS1-Melatonin | ALDH2^-/-^ | ALDH2^-/-^-APP/PS1 | ALDH2^-/-^-APP/PS1-Melatonin |
| --- | --- | --- | --- | --- | --- | --- | --- |
| Body Weight (BW, g) | 26.8 ± 0.5 | 26.5 ± 0.7 | 26.1 ± 0.6 | 26.4 ± 0.5 | 25.8 ± 0.6 | 25.2 ± 0.5 | 25.8 ± 0.6 |
| Heart Weight (HW, mg) | 146 ± 4 | 140 ± 3 | 148 ± 3 | 143 ± 3 | 139 ± 4 | 144 ± 4 | 137 ± 4 |
| HW/BW (mg/g) | 5.47 ± 0.12 | 5.30 ± 0.05 | 5.70 ± 0.17 | 5.41 ± 0.08 | 5.39 ± 0.10 | 5.69 ± 0.07 | 5.32 ± 0.09 |
| Liver Weight (LW, g) | 1.41 ± 0.03 | 1.44 ± 0.04 | 1.42 ± 0.03 | 1.39 ± 0.03 | 1.37 ± 0.03 | 1.37 ± 0.03 | 1.34 ± 0.03 |
| LW/BW (mg/g) | 53.0 ± 1.8 | 54.3 ± 1.0 | 54.6 ± 1.6 | 52.8 ± 1.0 | 53.0 ± 0.6 | 54.4 ± 0.6 | 52.2 ± 0.8 |
| Kidney Weight (KW, g) | 0.374 ± 0.008 | 0.384 ± 0.025 | 0.371 ± 0.010 | 0.378 ± 0.014 | 0.391 ± 0.018 | 0.366 ± 0.024 | 0.374 ± 0.018 |
| KW/BW (mg/g) | 14.1 ± 0.4 | 14.4 ± 0.7 | 14.3 ± 0.5 | 14.3 ± 0.4 | 15.1 ± 0.4 | 14.4 ± 0.7 | 14.4 ± 0.4 |
| Glucose (mg/dl) | 101.9 ± 4.3 | 101.0 ± 4.0 | 95.2 ± 4.0 | 102.3 ± 3.9 | 100.7 ± 3.8 | 104.9 ± 3.2 | 100.4 ± 3.8 |

Data are shown as Mean ± SEM, n = 9-14 mice per group, p > 0.05 for all parameters among these mouse groups.

Supplemental Data Figure S1:

Supplemental Data Fig. S1: ALDH2 expression in heart tissue of WT and APP/PS1 mice treated with or without melatonin and PKCε inhibitor εV1-2. Neither melatonin nor εV1-2 affects ALDH2 expression. Data are shown as Mean ± SEM, n = 6 mice per group.

Supplemental Figure S2:

Fig. S2: The role of melatonin membrane receptor and autophagy on melatonin-mediated cardiac protection in WT, APP/PS1 and ALDH2^-/-^-APP/PS1 mice. a: MT1 expression in cardiac tissue; b: MT2 expression in cardiac tissue; c: Resting cell length of cardiomyocytes from WT and APP/PS1 mice treated with or without melatonin receptor antagonist luzindole (Luz, 5 μM) and autophagy inhibitor 3-MA (10 mM); d: Peak shortening (normalized to cell length); e: Maximal velocity of shortening (+dL/dt); f: Maximal velocity of relengthening (-dL/dt); g: Time-to–peak shortening (TPS); and h: Time-to-90% relengthening (TR_90_); Data are Mean ± SEM, n = 4-6 mice per group for panel a-b, n = 33 cells from 3 mice (10-12 cells per mice) for panel c-h. *p < 0.05 between the indicated groups.

Supplemental Figure S3:

Fig. S3: Effect of melatonin on inflammatory cytokine levels in heart tissue of WT, APP/PS1 and ALDH2^-/-^-APP/PS1 mice. a: TNF-α level; and b: IL-1β level. Insets: Representative gel blots depicting levels of TNF-α and IL-1β (GAPDH as loading control). Data are shown as Mean ± SEM, n = 5-8 mice per group. *p < 0.05 between the indicated groups.

Supplemental Figure S4:

Fig. S4: Co-Immunoprecipitation assay showing the possible interaction between ALDH2 and cGAS. Co-Immunoprecipitation assay of ALDH2 and cGAS in lysates from WT mouse heart.

Supplemental Figure S5:

Fig. S5: WT and APP/PS1 neonatal cardiomyocytes were transfected with GFP-LC3 adenovirus followed by silencing cGAS or STING with siRNA for 72 hrs prior to incubation with or without melatonin (MEL, 100 μΜ) for 4 hrs. a: Representative images of GFP-LC3, MitoTracker, DAPI staining and merged images in treated with or without melatonin, cGAS siRNA and STING siRNA; b: Quantitative analysis of GFP-LC3 and MitoTracker co-localized puncta per cell; c: Neonatal cardiomyocyte caspase-3 activity; d: Validation of RNA silencing of cGAS; and e: Validation of RNA silencing of STING. Data are shown as Mean ± SEM, n= 5 biological repeats, *p < 0.05 between the indicated groups.

**REFERENCES**

1 Turdi, S. et al. Cardiomyocyte contractile dysfunction in the APPswe/PS1dE9 mouse model of Alzheimer's disease. *PLoS One* **4**, e6033 (2009).

2 Zhang, M. et al. Melatonin protects against diabetic cardiomyopathy through Mst1/Sirt3 signaling. *Journal of pineal research* **63**, (2017).

3 Emanuele, E. et al. Can enhanced autophagy be associated with human longevity? Serum levels of the autophagy biomarker beclin-1 are increased in healthy centenarians. *Rejuvenation Res* **17**, 518-524 (2014).

4 Misaka, T. et al. Plasma levels of melatonin in dilated cardiomyopathy. *Journal of pineal research* **66**, e12564 (2019).

5 Qiu, L. Y. et al. Sasanquasaponin promotes cellular chloride efflux and elicits cardioprotection via the PKCepsilon pathway. *Molecular medicine reports* **13**, 3597-3603 (2016).

6 Pang, J. et al. Mitochondrial ALDH2 protects against lipopolysaccharide-induced myocardial contractile dysfunction by suppression of ER stress and autophagy. *Biochim Biophys Acta Mol Basis Dis* **1865**, 1627-1641 (2019).

7 Ali, T., Badshah, H., Kim, T. H. & Kim, M. O. Melatonin attenuates D-galactose-induced memory impairment, neuroinflammation and neurodegeneration via RAGE/NF-K B/JNK signaling pathway in aging mouse model. *Journal of pineal research* **58**, 71-85 (2015).

8 Wang, S., Zhu, X., Xiong, L. & Ren, J. Ablation of Akt2 prevents paraquat-induced myocardial mitochondrial injury and contractile dysfunction: Role of Nrf2. *Toxicol Lett* **269**, 1-14 (2017).

9 Guo, R., Hu, N., Kandadi, M. R. & Ren, J. Facilitated ethanol metabolism promotes cardiomyocyte contractile dysfunction through autophagy in murine hearts. *Autophagy* **8**, 593-608 (2012).

10 Zhang, Y. et al. Mitochondrial aldehyde dehydrogenase (ALDH2) protects against streptozotocin-induced diabetic cardiomyopathy: role of GSK3beta and mitochondrial function. *BMC Med* **10**, 40 (2012).

11 Ren, J. Paradoxical effects of pyruvate on cardiac contractile function under normal and high glucose in ventricular myocytes. *Pharmacol Res* **48**, 25-29 (2003).

12 Hall, J. et al. Discovery of PF-06928215 as a high affinity inhibitor of cGAS enabled by a novel fluorescence polarization assay. *PLoS One* **12**, e0184843 (2017).

13 Wang, S. et al. ALDH2 protects against high fat diet-induced obesity cardiomyopathy and defective autophagy: role of CaM kinase II, histone H3K9 methyltransferase SUV39H, Sirt1, and PGC-1alpha deacetylation. *Int J Obes (Lond)* **42**, 1073-1087 (2018).

14 Li, S. et al. The Cyclopeptide Astin C Specifically Inhibits the Innate Immune CDN Sensor STING. *Cell Rep* **25**, 3405-3421 e3407 (2018).

15 Pei, J. et al. STAT3 inhibition enhances CDN-induced STING signaling and antitumor immunity. *Cancer Lett* **450**, 110-122 (2019).

16 Clark, K., Plater, L., Peggie, M. & Cohen, P. Use of the pharmacological inhibitor BX795 to study the regulation and physiological roles of TBK1 and IkappaB kinase epsilon: a distinct upstream kinase mediates Ser-172 phosphorylation and activation. *J Biol Chem* **284**, 14136-14146 (2009).

17 Yu, L. et al. Melatonin rescues cardiac thioredoxin system during ischemia-reperfusion injury in acute hyperglycemic state by restoring Notch1/Hes1/Akt signaling in a membrane receptor-dependent manner. *Journal of pineal research* **62**, (2017).

18 Zhou, H., Yue, Y., Wang, J., Ma, Q. & Chen, Y. Melatonin therapy for diabetic cardiomyopathy: A mechanism involving Syk-mitochondrial complex I-SERCA pathway. *Cell Signal* **47**, 88-100 (2018).

19 Wang, S. et al. Ablation of toll-like receptor 4 attenuates aging-induced myocardial remodeling and contractile dysfunction through NCoRI-HDAC1-mediated regulation of autophagy. *J Mol Cell Cardiol* **119**, 40-50 (2018).

20 Mao, Y. et al. STING-IRF3 Triggers Endothelial Inflammation in Response to Free Fatty Acid-Induced Mitochondrial Damage in Diet-Induced Obesity. *Arterioscler Thromb Vasc Biol* **37**, 920-929 (2017).

21 Ren, J. et al. Inhibition of CYP2E1 attenuates myocardial dysfunction in a murine model of insulin resistance through NLRP3-mediated regulation of mitophagy. *Biochim Biophys Acta Mol Basis Dis* **1865**, 206-217 (2019).

22 Zhou, H. et al. Pathogenesis of cardiac ischemia reperfusion injury is associated with CK2alpha-disturbed mitochondrial homeostasis via suppression of FUNDC1-related mitophagy. *Cell Death Differ* **25**, 1080-1093 (2018).

23 Nakahira, K. et al. Autophagy proteins regulate innate immune responses by inhibiting the release of mitochondrial DNA mediated by the NALP3 inflammasome. *Nature immunology* **12**, 222-230 (2011).

24 Maekawa, H. et al. Mitochondrial Damage Causes Inflammation via cGAS-STING Signaling in Acute Kidney Injury. *Cell Rep* **29**, 1261-1273 e1266 (2019).

25 Yuan, L. et al. Palmitic acid dysregulates the Hippo-YAP pathway and inhibits angiogenesis by inducing mitochondrial damage and activating the cytosolic DNA sensor cGAS-STING-IRF3 signaling mechanism. *J Biol Chem* **292**, 15002-15015 (2017).
